# Prospective Analysis of Incisional Morbidity Associated With Anterior Surgical Approaches to the Lumbar Spine

**DOI:** 10.7759/cureus.64587

**Published:** 2024-07-15

**Authors:** Riza M Cetik, John R Dimar, Morgan E Brown, Christy L Daniels, Leah Carreon

**Affiliations:** 1 Orthopedics, Norton Leatherman Spine Center, Norton Healthcare, Louisville, USA; 2 Research, Norton Leatherman Spine Center, Norton Healthcare, Louisville, USA

**Keywords:** quality improvement, cosmesis, incisional morbidity, interbody fusion, anterior lumbar

## Abstract

Objective: Anterior approaches to the lumbar spine have been used extensively for various indications but they are also associated with unique complications and have been linked with higher incisional morbidity.This study aimsto evaluate incisional morbidity related to anterior lumbar surgeries and to assess how incisional outcomes correlate with patient and surgery-related factors.

Methods: Patients ≥18 years old and with planned anterior lumbar fusions from L1 to S1 were prospectively enrolled. Follow-up ended at two years, and patients who did not complete the follow-up were excluded. Incision was assessed for general appearance, width, color, cross-hatching, hypertrophy, and pain by using a validated scoring system and a visual analog scale (VAS). Patient and surgery-related factors were analyzed for possible correlations with complications or wound-related parameters.

Results: A total of 205 patients with a mean age of 54.4 ± 11.5 were included. Significant improvements were seen in color, hypertrophy, pain, and appearance of the incision. At two years, the mean patient-based VAS for appearance was 8.6 while surgeon-based VAS was 8.8. The total rate of complications was 9%, with no incisional hernia or bulging. No significant relation was found between incision-related parameters and the demographic and surgical variables.

Conclusion: This study reports acceptable cosmetic results and no chronic pain after anterior lumbar surgery, which is contrary to previous reports. Together with a low total rate of complications, anterior approaches are safe when carefully executed, and have low morbidity.

## Introduction

Anterior approaches to the lumbar spine have been used extensively for various indications, including deformity, degeneration, trauma, tumors, and infections [1]. Compared to the more commonly utilized posterior-based techniques, anterior approaches offer direct access to the anterior column allowing for potentially improved reconstruction of the spinal alignment and restoration of the structural integrity. There are also significant biomechanical benefits of anterior fusions, including a larger surface area for grafting, a wider endplate for interbody grafting, and reduced strain on the screws when combined with posterior operations [2]. Over the last two decades, technological and technical advancements led to an increase in the frequency of anterior lumbar surgeries: an average 24% increase in the annual number of anterior interbody fusions has been reported [3].

Anterior lumbar surgery is associated with unique complications that require specialized expertise. Intraoperative and approach-related complications are especially troublesome and include injuries to the major thoracic and lumbar vessels, bowel perforations, peritoneal tears and ileus, ureter lacerations, retrograde ejaculation, lymphatic injuries, contusions to the spleen and liver and incisional hernias, particularly with extensile incisions [4]. Despite the appealing advantages for a very wide range of pathologies, some surgeons have linked anterior lumbar surgeries with increased morbidity and worse functional outcomes [5,6]. These concerns caused some surgeons to advocate against anterior surgeries despite their potential significant advantages for certain pathologies that may be treated more effectively with this approach. With new techniques and forming dedicated teams including access surgeons, more recent studies reported acceptably low overall complication rates, leading to a resurgence of anterior lumbar surgery for the effective treatment of specific spinal pathologies [4,7,8].

Incisional morbidity is controversial for anterior lumbar surgeries, as Kim et al. pointed out in a series of patients with a very extensive lateral thoracolumbar incision [5]. Other surgeons have pointed out that anterior surgery in adult deformity results in longer operative times, more blood loss, higher costs, and increased incisional morbidities [6]. This does not negate the important requirement that anterior surgery is extremely useful and provides an important alternative for certain pathologies such as fractures, tumors, osteomyelitis, and appropriate deformities. New minimal incisional techniques are now used in the majority of patients undergoing anterior lumbar surgery, which has changed the possible risk for injuries to the abdominal muscles and the transection of the T11 and T12 intercostal nerves that innervate the abdominal muscles. This problem, which results in troublesome abdominal injuries, has been a frequent complaint expressed by both patients and their operating surgeons [5,6,9].

This prospective study evaluates incisional morbidity related to anterior lumbar surgeries and assesses how incisional outcomes correlate with patient and surgery-related factors. We hypothesize that carefully executed traditional and regional mini-anterior lumbar surgeries preserve the anterior abdominal wall fascia, muscular layers, and innervation, resulting in minimal incisional morbidity.

## Materials and methods

This study was designed as a prospective longitudinal cohort study. Written informed consent was obtained from all participating patients. The EQUATOR Network reporting guidelines based on the Strengthening the Reporting of Observational Studies in Epidemiology (STROBE) checklist were used for the preparation of this study. After receiving Institutional Review Board approval from the University of Louisville (HSPPO#14.0567/RO#14.N0125) patients were prospectively enrolled at a single center between June 2013 and December 2017. Inclusion criteria included patients ≥18 years old, who underwent planned anterior lumbar fusions from L1 to S1, and patients who signed informed consent. Patients were followed up for two years after the surgery. Utilized incisions were lateral/flank muscle splitting retroperitoneal, paramedian retroperitoneal, oblique anterolateral muscle splitting incision, rectus sparing, and transabdominal (either Pfannenstiel or midline vertical) [2]. None of the cases were minimally invasive surgeries. No topical antibiotics were used in the anterior incision. Two surgeons were involved in all the cases with a vascular surgeon performing the approach and the spine surgeon performing the rest of the procedure and wound closure.

Demographic data included age, gender, body mass index (BMI), and smoking status. Surgical data included operative time, estimated blood loss (EBL), number of fused levels, surgical approach utilized, indications, American Society of Anesthesiologists (ASA) scores, prior abdominal and spinal surgeries, complications, and length of hospital stay (LOS). Incisional data included width, color, cross-hatching, and hypertrophy by utilizing a scoring system proposed by Trimbos et al. and validated in several studies (Table 1) [10,11]. Each variable is rated between 1 to 4, with higher numbers indicating worse outcomes. A visual analog scale (VAS) [12,13] was used for rating the appearance of the incision (with 0 being the worst and 10 being the best cosmetically) and incisional pain (with 0 being none and 10 being unbearable). Data for this study were collected from the follow-up visits at postoperative week one (as baseline), 6th month, 12th month, and 24th month.

**Table 1:** Wound-related parameters collected during the study period.

Grade	Hypertrophy	Grade	Width (mm)
1	No elevation above the surrounding skin	1	<1 mm
2	Minimum elevation	2	1-2 mm
3	Hypertrophic but acceptable	3	3-5 mm
4	Distinct hypertrophy	4	>5 mm
Grade	Color	Grade	Cross-hatching
1	No difference from the surrounding skin	1	Absent
2	Minimal difference	2	Slightly visible
3	Marked difference	3	Clearly present
4	Purple blue scar, unacceptably different	4	Unacceptable

Continuous data are presented as means with standard deviations, and frequencies are presented with percentages. To confirm a normal distribution, Shapiro-Wilk’s test, histograms, skewness/kurtosis calculations, and detrended Q-Q plots were used. To compare the outcome variables at different time points, repeated measures ANOVA or Friedman test was used. Correlations between variables were assessed by using Pearson and Spearman tests. Statistical analyses were performed using SPSS v26 (IBM Corp., Armonk, NY). Statistical significance was set at p = 0.05.

## Results

Of the 292 patients undergoing anterior lumbar surgery enrolled in the study, 205 (70%) with two-year follow-up were included. Eighty-seven patients were excluded for not completing the two-year follow-up period. The main demographic and surgical parameters are summarized in Table 2.

**Table 2:** Demographic and surgical parameters of the study population.

Variable	Study population (n = 205)
Age (mean ± SD)	54.4 ± 11.5
Gender (M/F)	104/101
Body mass index (kg/m^2^, mean ± SD)	31.7 ± 7.1
Smoking status (n, %)	53 (26%)
Prior abdominal surgery (n, %)	88 (43%)
Prior anterior spine surgery (n, %)	13 (6%)
Prior posterior spine surgery (n, %)	101 (49%)
Indications for surgery (n, %)	
Spondylolisthesis	53 (26%)
Stenosis	46 (22%)
Non-union	34 (17%)
Mechanical disc collapse	20 (10%)
Adjacent degeneration	18 (9%)
Scoliosis	12 (6%)
Osteomyelitis	11 (5%)
Fracture	5 (2%)
Post-discectomy	4 (2%)
Recurrent herniation	2 (1%)
Number of fused levels (n, %)	
1	111 (54%)
2	68 (33%)
3	21 (10%)
4	3 (1%)
5	2 (1%)

The majority of patients involved fusion at L5-S1 (39%), followed by fusion at L4-S1 (23%). Mean LOS was 6.5 ± 3.5 days, operative time was 237 ± 141 minutes, and EBL was 15.2 ± 45.0 ml. Most frequently used approach was the lateral/flank muscle splitting retroperitoneal (49, 24%), paramedian retroperitoneal (41, 20%), Pfannenstiel (transabdominal) (39, 19%), oblique anterolateral (31, 15%), vertical midline (transabdominal) (27, 13%), transverse (12, 6%), and rectus sparing (6, 3%). The length of the incision was similar among the different approaches (p = 0.623, Table 3).

**Table 3:** Length of surgical incision stratified by approach.

Approach	N (%)	Incision length, cm, mean (SD)
Lateral/flank muscle splitting retroperitoneal	49 (24%)	156.14 (35.89)
Paramedian retroperitoneal	41 (20%)	118.28 (25.40
Pfannenstiel/transabdominal	39 (19%)	135.97 (24.81)
Oblique anterolateral	31 (15%)	117.77 (30.44)
Vertical midline/transabdominal	27 (13%)	155.25 (37.90)
Transverse	12 (6%)	159.07 (57.58)
Rectus sparing	6 (3%)	115.45 (4.54)

Changes in the incision-related parameters are summarized in Table 4. Significant improvements were seen in color, hypertrophy, pain, and appearance of the incision scar during the follow-up period. Only five (2%) patients noted a painful (VAS > 4) scar at the 24-month follow-up, almost all due to a hypertrophic scar. Although statistically significant correlations were found between some incision-related parameters and the demographic and surgical variables, all of the associations were weak (<0.5). Included variables in this analysis were age, BMI, smoking status, preoperative ASA score, prior abdominal, anterior lumbar, or posterior lumbar spine surgeries, presence of diabetes, number of instrumented levels, surgical time, estimated blood loss, and the specific surgical approach chosen (Table 5). There were 18 (9%) complications in 16 (8%) cases (Table 6).

**Table 4:** Changes in wound-related parameters during the study period. P-value based on repeated measures ANOVA for within-subject differences. A p-value less than 0.01 is considered to be statistically significant. VAS: visual analog scale.

Wound-related parameters (mean ± SD)	Baseline	6 months	12 months	24 months	p-value
Color	2.03 ± 0.73	1.82 ± 0.64	1.71 ± 0.59	1.63 ± 0.71	0.011
Hypertrophy	1.50 ± 0.67	1.29 ± 0.77	1.27 ± 0.75	1.07 ± 0.51	0.000
Width	1.51 ± 0.68	1.68 ± 0.57	1.54 ± 0.70	1.36 ± 0.63	0.093
Cross-hatch	1.35 ± 0.64	1.21 ± 0.57	1.15 ± 0.63	1.09 ± 0.42	0.128
Pain	2.46 ± 2.05	1.81 ± 1.59	1.46 ± 1.09	0.96 ± 0.87	0.032
Length (mm)	137.05 ± 3.08	136.03 ± 2.85	134.96 ± 2.57	135.13 ± 2.00	0.328
Appearance (patient-based VAS)	7.33 ± 2.86	7.81 ± 2.05	8.01 ± 1.92	8.60 ± 2.32	0.041
Appearance (surgeon-based VAS)	7.44 ± 2.93	8.21 ± 1.70	8.60 ± 1.99	8.84 ± 1.34	<0.001

**Table 5:** Correlation coefficients between patient and surgical variables and surgical scar parameters at the 24-month follow-up. ** Correlation is significant at the 0.01 level (two-tailed). * Correlation is significant at the 0.05 level (two-tailed). ASA: American Society of Anesthesiologists.

	Surgical scar parameters							
Variable	Color	Hypertrophy	Width	Cross-hatching	Pain	Length	Appearance (patient)	Appearance (surgeon)
Age	-0.214	-0.457**	-0.04	0.07	-0.115	0.147	0.18	0.321*
BMI status	0.353**	0.431**	0.292*	0.015	-0.04	0.174	0.143	-0.359**
Smoking status	0.013	-0.059	0.067	-0.149	-0.031	-0.133	-0.127	0.014
ASA	0.184	0.14	0.133	-0.147	0.132	-0.045	-0.027	-0.19
Diabetes	-0.018	-0.015	-0.093	-0.007	0.029	-0.004	0.023	-0.029
Number of levels	0.097	-0.055	0.096	0.14	0.116	0.291*	-0.174	-0.169
Prior abdominal surgery	0.02	-0.072	-0.131	-0.065	-0.174	-0.169	-0.132	0.071
Prior anterior spine surgery	-0.227	-0.067	-0.15	-0.134	-0.081	-0.228	-0.142	0.15
Operative time	0.310*	0.194	0.215	-0.04	0.021	-0.015	-0.271*	-0.246
Estimated blood loss	0.233	0.399**	0.089	-0.154	-0.044	0.279*	-0.021	-0.217
Surgical approach	-0.059	0.002	-0.164	0.024	-0.055	0.06	0.079	0.082

**Table 6:** Complications documented during the study period.

Complications	n (%)
Incisional hernia	0
Incisional bulging	0
Deep infection	2 (1%)
Superficial infection	2 (1%)
Ileus	7 (3%)
Deep vein thrombosis	3 (1%)
Pulmonary embolism	0
Retrograde ejaculation	0
Vascular injury	0
Visceral injury	0
Radiculopathy	4 (2%)

## Discussion

On a prospective analysis of 205 patients, we found that the incisional morbidity and cosmetic results of anterior lumbar surgical incisions significantly improved after a follow-up of two years. With good cosmetic results and no chronic pain, this study shows that anterior lumbar surgery has low incisional morbidity contrary to previously reported findings. Together with an acceptable rate of complications, surgeons can safely rely on these procedures and keep them in their armamentarium.

Outcomes of anterior lumbar spine surgery have been evaluated by many different studies [4-6,14,15]. The literature has highlighted that incisional problems are often a source of patient discomfort and complaints due to abdominal hernias. In one of the first studies to put an emphasis on this, Kim et al. reported in 2009 that anterior lumbar surgery was burdened with a very high rate of incisional complications, and 61% of the patients had at least one type of complication on the incision site [5]. Of the patients, 32% reported chronic pain at the incision site, and a majority of these patients reported no improvements at five and 10 years postoperatively. It must be noted that in this study, all of the patients were treated by using an anterolateral flank incision, which is more extensile and traumatic than most of the other anterior approaches. Fischer et al. also reported 35% pain around the anterior lumbar incision scar, which did not improve after the first year for 25% of the patients, but the mentioned study did not include detailed information on the surgical approaches [15]. Another potential drawback of the anterolateral flank incision is that the intercostal nerves (T11 and T12) cross the surgical field within the abdominal musculature, which brings the risk of intraoperative nerve injury or post-surgical entrapment that may result in neuropathic pain [2,16]. Jagannathan et al. focused on comparing the functional and cosmetic outcomes of anterolateral retroperitoneal and anterior paramedian approaches: patients who had an anterior paramedian approach scored significantly better at Scoliosis Research Society 22-Item (SRS-22) pain (4.4 vs. 3.2, p < 0.001), self-image (4.5 vs. 3.9, p = 0.004), and functional activity scores (4.2 vs. 3.1, p = 0.003) [9]. However, no difference was found in the VAS scores. This study does not support many of the findings of certain previous studies when using a specific incisional outcome measure. Incision color, hypertrophy, and pain showed significant improvements in two years. In the 2nd year of follow-up, the mean incisional pain score was 0.96, which is close to an excellent outcome. We also did not find a significant relationship between any of the wound scar scoring system subdomains and specific surgical approaches. We performed the correlations analysis based on the 2nd year data and the overall pain scores were very low, therefore very subtle differences, even if they do exist, may not have been detected.

The cosmetic results we reported were favorable, with mean scores of less than 2 in each subdomain. Mean VAS wound appearance scores were also between 8 and 9 for both patient and surgeon-based assessments. Observations of Jagannathan et al. were similar, and if the patients with incisional bulging were separated, both the paramedian and anterolateral incision groups scored between less than 2 on the same wound assessment scale, and the VAS scores (0-100) were over 80 [9]. Postoperative bulging and incisional hernia are closely related to persistent pain, cosmetic dissatisfaction, and abdominal stiffness [15,17,18]. This complication has been frequently reported after different surgical procedures involving flank incisions and is almost always linked with poor outcomes [19,20]. We reported no incisional hernias and no abdominal wall bulges, and we believe that this highly contributed to our positive outcomes. Prevention of these complications lies in exercising meticulous surgical techniques: avoiding extensive splitting and denervation of the muscles and direct repair of the fascia and all the muscular layers are of utmost importance. Another very important factor we feel is to establish a combined surgical team consisting of a spine surgeon and a vascular/general surgeon [8].

In the current study, no correlations were seen between the incisional outcome measures and patient or surgery-related factors. Previous studies suggested otherwise. BMI > 30 was linked with increased pain at the incision site [15] and overall complication rate [21]. Prior abdominal surgery has also been linked with a 49% rate of operative complications, particularly if a revision of the previous anterior lumbar surgery is performed [22]. It must be noted, however, that Jagannathan et al. did not find prior abdominal surgery to be related to postoperative bulging [9]. Larger studies are required to elaborate on these relations.

This study has certain limitations. First, it included four different surgical approaches, which bring heterogeneity and may confound the results. However, this reflects what we feel is the relative spectrum of anterior surgical approaches required by a spine surgeon to address all of the different pathologies and levels of involvement they would typically encounter when treating patients, i.e., there is no approach that can be universally used. We know that some complications tend to be seen more frequently with certain approaches (e.g., anterolateral flank incision-abdominal bulging), but these can be generally avoided by preserving the abdominal innervation and fascia planes. Second, the two scoring systems we used for scar assessment may not be suitable for extrapolation. The wound scale was specifically designed for abdominal wounds and has subdomains that address the most important aspects of wound cosmesis, but its use in the literature is very limited. The VAS score is widely used and has shown good inter- and intraobserver agreement on healed scars [23]. However, it is not standardized and the parameters assessed may vary in each study [24]. Third, associations between complications and the number of surgical levels could not be evaluated due to the low incidence of complications. Fourth, functional outcomes were not assessed in this study. Some questionnaires, such as the SRS-22 revised (SRS-22r), include questions about self-image and could contribute to our results on cosmesis [25].

## Conclusions

In conclusion, this prospective study demonstrates that in contrary to prior reports, carefully executed anterior lumbar approaches that preserve the anterior abdominal wall fascia and muscular layers along with the abdominal wall’s innervation results in successful outcomes with low incisional morbidity at two years follow-up. Previously reported chronic incisional pain and laxity problems are not unavoidable, and anterior lumbar spine surgery can be performed safely.
